# Assessing brain-muscle networks during motor imagery to detect covert command-following

**DOI:** 10.1186/s12916-025-03846-0

**Published:** 2025-02-06

**Authors:** Emilia Fló, Daniel Fraiman, Jacobo Diego Sitt

**Affiliations:** 1 Sorbonne Université, Institut du Cerveau - Paris Brain Institute - ICM, INSERM, CNRS, Paris, France; 2 Departamento de Matemática y Ciencias, Universidad de San Andrés, Buenos Aires, Argentina; 3 CONICET, Buenos Aires, Argentina

**Keywords:** Motor imagery, EMG, EEG, ECG, Brain-muscle networks, Disorders of consciousness

## Abstract

**Background:**

In this study, we evaluated the potential of a network approach to electromyography and electroencephalography recordings to detect covert command-following in healthy participants. The motivation underlying this study was the development of a diagnostic tool that can be applied in common clinical settings to detect awareness in patients that are unable to convey explicit motor or verbal responses, such as patients that suffer from disorders of consciousness (DoC).

**Methods:**

We examined the brain and muscle response during movement and imagined movement of simple motor tasks, as well as during resting state. Brain-muscle networks were obtained using non-negative matrix factorization (NMF) of the coherence spectra for all the channel pairs. For the 15/38 participants who showed motor imagery, as indexed by common spatial filters and linear discriminant analysis, we contrasted the configuration of the networks during imagined movement and resting state at the group level, and subject-level classifiers were implemented using as features the weights of the NMF together with trial-wise power modulations and heart response to classify resting state from motor imagery.

**Results:**

Kinesthetic motor imagery produced decreases in the mu-beta band compared to resting state, and a small correlation was found between mu-beta power and the kinesthetic imagery scores of the Movement Imagery Questionnaire-Revised Second version. The full-feature classifiers successfully distinguished between motor imagery and resting state for all participants, and brain-muscle functional networks did not contribute to the overall classification. Nevertheless, heart activity and cortical power were crucial to detect when a participant was mentally rehearsing a movement.

**Conclusions:**

Our work highlights the importance of combining EEG and peripheral measurements to detect command-following, which could be important for improving the detection of covert responses consistent with volition in unresponsive patients.

**Supplementary Information:**

The online version contains supplementary material available at 10.1186/s12916-025-03846-0.

## Background

Disorders of consciousness (DoC) refers to a group of pathological states in which consciousness is affected as a result of injury or trauma to the nervous system. Unresponsive wakeful syndrome also referred to as vegetative state (VS/UWS) and minimally conscious state (MCS) are two distinct categories of states of impaired consciousness. Patients in VS/UWS are awake (intermittent eye-opening) without reproducible volitional behavior [1, 2], in contrast, patients in MCS can occasionally show overt signs of awareness or responses to sensory stimuli beyond reflexes [3], suggesting cortically mediated behavior [4]. In order to classify patients with DoC in one of these states, clinical assessments are carried out to examine voluntary behaviors [5]. Accurately distinguishing patients in VS/UWS from patients in MCS based on behavioral criteria is a challenging task. Indeed, it is estimated that 40% of patients with DoC are incorrectly classified as VS/UWS [6, 7]. The level of conscious awareness of a patient can be underestimated as a consequence of fluctuations in arousal, difficulty in identifying behavior consistent with volition [8], as well as patients’ impossibility to convey overt responses due to medication, and sensory, or motor lesions [9]. Importantly, the diagnosis has a great impact on treatment, prognosis, and end-of-life decisions, as patients in MCS have a higher probability of regaining cognitive function [10–13]. Therefore, developing sensitive, precise, and objective tools to measure volitional behavior is of the highest clinical and ethical interest.

### Command-following beyond behavior

In addition to behavioral evaluation, non-invasive neuroimaging techniques such as functional magnetic resonance imaging (fMRI) and electroencephalography (EEG) have been widely used to assess the level of awareness of patients with DoC. The rationale is that if a patient is faced with a task that requires conscious processing and their brain response is sustained and consistent with the one elicited for healthy controls, then consciousness may be inferred [14, 15]. Motor imagery (MI) has been a recurrently used paradigm since Owen’s seminal study, where fMRI together with complex imagery tasks, such as navigating space and playing sports, enabled the identification of sustained command-following responses in a patient clinically diagnosed as in VS/UWS [16]. MI can be defined as a dynamic mental state during which there is a rehearsal of a motor act without overt body movement [17, 18]. MI can be divided into kinesthetic imagery, a first-person process that requires one to “feel” the movement or reproduce the sensations that the muscle contractions would produce, and visual imagery, a third-person perspective where one sees oneself performing the movement [19]. In practice, the term MI is widely used to refer to the first-person experience [20]. Numerous studies have found covert responses in VS/UWS and MCS patients using complex imagery paradigms (see [21, 22] for a review). Although with variable sensitivity, these studies managed to detect covert responses in MCS patients, and crucially in a few VS/UWS patients, illustrating that the information provided by these evaluations is a significant complement to bedside examinations. Nevertheless, some patients that have overt responses to commands fail to show a modulation of brain activity during these imagery tasks [23–26]. The high cognitive demands associated with these events could explain the observed inconsistency. Preserved cognitive functionalities among DoC patients are highly variable as brain injuries are commonly accompanied by other disorders or pathologies, consequently, complex tasks may in some cases underestimate the level of awareness [27]. Simpler motor tasks have been shown to be effective in detecting covert responses in VS/UWS patients [27], and in some cases they even provide a more accurate measure of command-following than complex motor imagery [28], suggesting they could be more appropriate to probe these patients.

### Covert responses measured with EEG in patients with DoC

EEG is low-cost, accessible in all health centers, suitable for all patients, and has proven effective to identify covert responses in DoC patients. Power modulations at various frequency bands and channels have been reported in MCS patients instructed to imagine swimming [29] or a sport of the patient’s choice [30]. Although variable, EEG responses have been detected in VS/UWS and MCS patients when asked to imagine opening and closing their hands [25, 31, 32] and moving their toes [31, 32]. EEG has also been combined with simple motor execution commands to evaluate DoC patients’ awareness levels. Demanding patients to move their feet on cue resulted in an EEG response in 2/6 MCS patients [30]. In a study comprising 104 unresponsive patients, instructions to open and close their hands elicited a power modulation in 16 individuals. Furthermore, responsive patients had better long-term outcomes than unresponsive patients [33]. This illustrates that EEG is an adequate tool to detect covert motor execution and motor imagery in non-communicative patients.

### Covert responses measured with EMG in patients with DoC

A less explored approach to evaluate awareness in patients without explicit motor or verbal responses is the use of surface electromyography (EMG). Research combining surface EMG with simple motor commands has shown subthreshold motor activity in DoC patients that failed to exhibit overt motor behavior. In a small study, one patient diagnosed as VS/UWS and one as MCS showed covert motor responses detected by EMG when asked to move their hands [34]. A subsequent study on a bigger cohort of patients and using multiple motor instructions showed EMG responses for a patient in VS/UWS and three patients in MCS [35]. Finally, using single-trial analyses of EMG activity, covert responses were detected in MCS patients when instructed to move both their right and left hands [36]. Therefore, muscular activity evaluated with a generally available and non-invasive tool such as surface EMG can provide meaningful information on a patient’s level of awareness.

### Corticomuscular coupling

Synchronization between muscle and cortical activity, typically referred to as corticomuscular coherence (CMC) [37], arises mainly from the primary motor cortex contralateral to the activated muscle, is somatotopically organized and its magnitude is directly correlated to the extent of the muscle cortical representation [38–41]. Coupling between EEG and EMG is typically found in the beta band (10–30 Hz) during weak contraction [42–45], is maximum when muscle contraction is stable, and disappears during movement [46] or movement preparation [47, 48]. At lower frequencies (< 10 Hz), and mainly during movement, corticokinematic coupling takes place elicited by afferent sensory information and movement rhythmicity [49]. Additionally, CMC in the gamma band (31–45 Hz) has been reported [42, 50, 51]. It is believed that the cortical mu rhythm contributes to the CMC at ~ 20 Hz [45, 52]. Mu rhythm is a sensorimotor oscillation that arises from a mixture of frequencies with different neurophysiological origins. Frequencies surrounding the alpha band (mu-alpha ~ 10 Hz) are considered to reflect activity from the somatosensory cortex while frequencies around the beta band (mu-beta ~ 20 Hz) would reflect motor cortex activation [53, 54]. The modulation of this rhythm by a movement event is referred to as event-related synchronization (ERS) when an increase of power is observed, or event-related desynchronization (ERD) when a decrease occurs [54–56]. Normally, a few seconds before initiation and during movement an ERD is observed followed by a rebound ERS when execution is stopped [46, 54, 57].

### Brain and body response during imagery movement

EEG response during motor imagery is variable such that a percentage of participants do not show this pattern [58–60]. Nevertheless, when present, MI shows similar brain activity signatures to motor execution (ME) (see [61] for a review). Although weaker, ERD of the mu rhythm occurs immediately before [62, 63] and during MI [59, 64, 65] followed by an ERS initiated by movement termination [60]. In addition to brain activity, bodily signals can serve as a window to detect mental processes. The autonomic nervous system (ANS) elicits changes to maintain bodily homeostasis and to prepare for environmental as well as internal demands. It has a close relationship to processes such as emotion and attention [66] and is also modulated by mental representations of movement, both observed and imagined. Kinesthetic imagery requires accessing stored information related to the sensations elicited by proprioceptors and exteroceptors during actual movement and movement preparation [67]. In this line, evidence shows an increase in heart rate during motor imagery with a magnitude of the effect related to the level of effort of the imagined movement [68–71], suggesting that the brain is in fact evoking an internal model of the movement comprising its metabolic demands [67]. On the other hand, evidence of covert contraction of the muscles during MI is not as robust (see [72] for a review). Some studies for which brain responses during MI are consistent with movement show no EMG response [69, 73–76], while studies for which subthreshold EMG activity is elicited, show that the response is correlated to the level of effort imagined [77, 78] and the muscles activated are consistent with the muscles involved in the mentally represented movement [79, 80]. Crucially, there is evidence of an increase in the excitability of descending motor pathways during MI tasks [81–85].

### Functional muscle networks

The musculoskeletal system is characterized by having a great number of degrees of freedom which makes it a very flexible but complex system [86]. Movement execution, as well as the mental rehearsal of a movement, requires the coordinated action of cortical and subcortical structures in space and time. Muscle synergies have been proposed as the strategy the nervous system has to simplify motor control while ensuring proper motor outputs [87, 88]. Muscle synergies can be defined as the coherent activation in space or time of a group of muscles orchestrated by motor areas of the cortex and the afferent systems [89]. In order to explore motor organization, intermuscular coherence (IMC) is computed as the cross-correlation in the frequency domain of the EMG response for each pair of muscles [90], and muscle synergies are identified by applying non-negative matrix factorization (NMF) [91] to the IMC spectra. Recently, a new approach that combines NMF and network analysis shows that muscle groups show coupling at different frequencies for different movements, postulating a functional organization of the muscle synergies which they refer to as functional muscle networks (FMN) [92, 93]. Experimental evidence suggests that muscle synergies derive from common neural input [94–96]. Therefore, FMN combined with corticomuscular coherence analysis could provide information on cortical descending control [97].

### This study

Finding markers of concealed command-following in healthy participants is the first step to developing new diagnostic tools for unresponsive patients. Moreover, markers based on equipment that is broadly available in any hospital are of particular importance. In this line, although muscular activity measured with surface EMG has shown some promising results, its potential to detect covert responses remains relatively unexplored. In this study, we propose to evaluate the potential of a network approach to electromyography and electroencephalography recordings to detect covert command-following in healthy participants. We will study the brain and muscle functional network configuration during motor execution, motor imagery, and resting state, and we will test the following hypothesis. (H1) The brain-muscle networks of healthy participants during motor imagery are different from the resting state networks and engage the same muscles as during motor execution. (H2) Autonomic responses are modulated by motor imagery. (H3) Subjects’ cortical responses during motor imagery are correlated to their motor imagery ability. (H4) Brain-muscle networks together with ERD/ERS and cardiac activity can provide information on covert command-following at the subject level.

The results of this investigation will determine the feasibility of applying this paradigm in the clinical context to detect persistent covert awareness in unresponsive patients.

## Methods

The task has been conceived with the underlying intention of a future application in the clinical context, and this has determined its design. It is a simple auditory task that requires non-invasive equipment commonly available in health centers that would cause minimal discomfort to patients. Moreover, it is inspired by a similar task that has already been shown to be effective in detecting covert awareness in patients with disorders of consciousness using EEG [33]. If the approach proposed in this work yields positive results on a healthy cohort of participants, it will be important to evaluate improvements in the protocol that would allow a more effective implementation in medical settings (see analysis section).

## Experimental procedure

### Motor imagery scale

Participants were evaluated on their ability to imagine movements. To this aim, the French-validated Movement Imagery Questionnaire-Revised Second version (MIQ-RS) [98] was carried out. On top of evaluating participants’ motor imagery ability, this scale served to exercise participants’ kinesthetic imagery for the upcoming task.

### Task

The following commands were presented to the participants:

1. Open and close your hand
2. Flex and extend your foot
3. Open and close your hand and flex and extend your foot
4. Imagine opening and closing your hand
5. Imagine flexing and extending your foot
6. Imagine opening and closing your hand and flexing your foot
7. Stay relaxed without moving or tensing your body

**Fig. 1:**
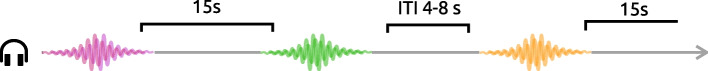
Experimental design. Each instruction is presented auditorily. Participants have to execute a movement, imagine a movement, or remain relaxed according to the received instruction for the duration of the trial. A stop command is presented 15 s after the initial instruction. Trials are separated by a random interval between 4 and 8 s

Participants had to repeat the requested action for 15 s until a stop command was heard. The task is structured in four blocks. In each block, each motor condition is presented 6 times and the resting condition is presented 18 times (54 trials per block), resulting in 72 trials of motor execution, 72 trials of motor imagery, and 72 trials of resting state. The conditions within each block are presented in a randomized fashion. Participants were asked to focus on the sensations during movement execution and to try to remember them when engaging in kinesthetic motor imagery. Instructions were presented binaurally through Etymotic ER3C Tubal Insert Earphones and participants were asked to remain with their eyes closed during the blocks. The inter-trial interval was randomly varied between 4 and 8 s (Fig. 1). A custom-built Arduino stimulation box was used to send the audio instructions and the event markers directly to the amplifier.

### Physiological recordings

Participants were seated in a high Fowler’s position and surface EMG electrodes were applied on their dominant hand. In addition, a bipolar electrode placed in the left and right collarbone was used to record cardiac activity. The skin was prepared by scrubbing with alcohol swabs in order to reduce the impedance and improve the contact between the skin and the electrodes. High-density EEG was recorded using EGI 256 channels HydroCel GSN net and EMG and ECG activity was recorded using the Physio16 MR input box. All signals were acquired with a Net Amps 400 EEG Amplifier from Electrical Geodesics, Inc, digitized at 1000 Hz.

### Preprocessing

Preprocessing for EMG and EEG data was carried out using MNE 1.0.3 [99] and Scipy 1.8.1 [100]. MNE and Scipy are open-source Python-based libraries. MNE is dedicated to the analysis of EEG and MEG signals, and much of its code is based on Scipy functionalities, a scientific and numerical tools library.

### EMG and intermuscular coherence

EMG data preprocessing followed the steps detailed in Kerkman et al. [93]. Briefly, the EMG signal was downsampled to 500 Hz and band-pass filtered 0.5–200 Hz (one-pass zero-phase FIR filter with length 6601 samples). The ECG artifact was removed using independent component analysis (ICA) and a 20 Hz high-pass filtered (one-pass zero-phase FIR filter with a length of 661 samples) was applied. Data was epoched from movement onset to stop command. Trials for which EMG activity in any channel was above 3 standard deviations of the mean in a condition and channel-specific manner were rejected. For this, the mean EMG activity for each channel and condition was computed. If for a given trial, EMG activity exceeded the threshold specific to that condition and channel for a minimum of 3 s of data, the trial was rejected. In order to ensure no overt movement during the motor imagery conditions, on top of the described criterion, we rejected a motor imagery trial if any channel exhibited EMG activity surpassing the average activity observed for that channel during the corresponding motor condition (i.e., EMG activity during “open and close your hand” conditions was used to assess overt movement during “Imagine opening and closing your hand” conditions), for a duration of at least 1 s of data. The data was rectified using the Hilbert transform and demodulated to remove slow fluctuations due to movement [101]. For each subject and trial, power spectral density was estimated using Welch’s periodogram method with a hamming window of 750 ms and an overlap of 550 ms with an fft length of 3 s. Before computing complex value spectral coherence, the autospectra was smoothed [102]. Complex value spectral coherence was obtained for each muscle pair and averaged across trials within each condition for group analysis. The absolute value of the resulting coherence was squared yielding a magnitude squared coherence (MSC) value per subject, muscle, and condition. In order to increase the number of trials for subject-level classification, each 15 s epoch was divided into 3 s epochs, yielding 5 sub-epochs per trial, and MSC was obtained for each one.

### EEG, corticomuscular coherence, and cortical coherence

EEG signals were bandpass filtered 0.5–40 Hz (one-pass zero-phase FIR filter with length 6601 samples). Electrodes over facial muscles were discarded. ICA will be applied to remove cardiac and eye movement artifacts. Bad channels were interpolated using Autoreject [103] and data was referenced to the average of the electrodes before epoching from the onset of movement to the stop command (0:15 s). The high-density EEG was reduced to 64 channels by interpolating neighboring channels. The decision to reduce EEG data to fewer sensors is motivated by trying to reduce the number of features fed to the classifiers (see below); this optimizes computational time while increasing the observations and features ratio. Following Roeder et al. [104], a bipolar montage was used to measure left (LSM: C3-F3 electrodes) and right (RSM: C4-F4 electrodes) sensorimotor activity. CMC was computed between C4-F4 and each muscle, and between C3-F3 and each muscle, following the same steps as to obtain intermuscular coherence. Cortical coherence (CC) was assessed between LSM and RSM using imaginary coherence to avoid pseudo-connectivity due to volume conduction [105]. For subject-level analyses, the same procedure as for EMG data was carried yielding 5 sub-epochs per trial and Autoreject was used to reject noisy sub-epochs.

### Brain-muscle networks

**Fig. 2:**
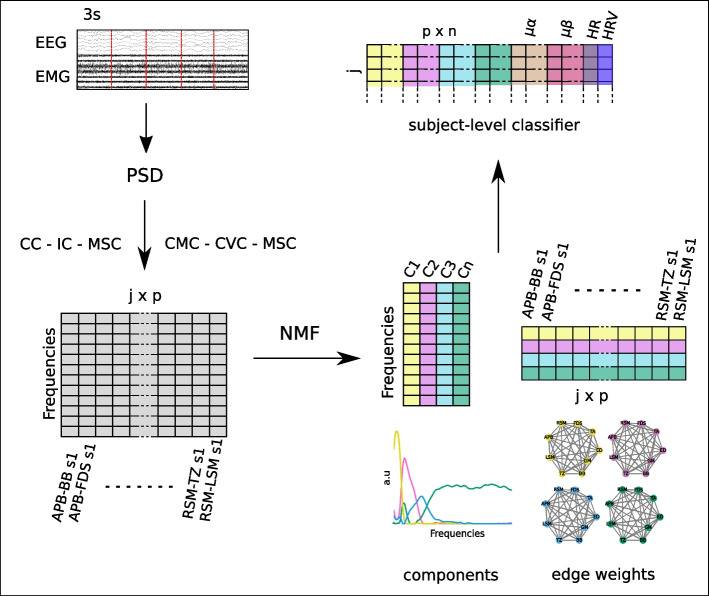
Feature extraction and subject-level classifiers. Each 15 s trial is subdivided into 3 s sub-epochs. Power spectral density is obtained for each sub-epoch. For corticomuscular coherence and muscle coherence, complex value spectral coherence (CVC) is computed for each EMG-EMG and EEG-EMG pair (p). The absolute value of the resulting coherence is squared yielding a magnitude squared coherence (MSC) value per sub-epoch and channel pair. In order to avoid spurious connectivity for cortical coherence, imaginary coherence (IC) is computed for the EEG channel pair (RSM − LSM). The resulting coherence matrix has dimensions sub-epochs × number of channel pairs (j × p). Non-negative matrix factorization is used to decompose the coherence matrix. The decomposition results in two matrices, one corresponding to frequency components and one to the weights for the different components. The frequency components matrix is a unique matrix whose final dimensions depend on the reconstruction quality of the original matrix evaluated with the Frobenius norm (n). The weights matrix can be represented as frequency-specific networks for each condition and component where the strength of connectivity for each pair of channels gives the edges of each network (in the figure the networks would correspond to the connectivity for each frequency component for a given condition). For classification purposes, a feature matrix is constructed such that for each sub-epoch the edge weights for each channel pair and frequency component are combined with the heart rate, heart rate variability during the 15 s epoch, and the power for the mu-alpha and mu-beta bands for the 64 channels during the sub-epoch. For group-level networks, the coherence values for each subject are averaged across the same condition trials before computing MSC, and therefore the resulting matrix has dimensions number of subjects × channel pairs × conditions, and the NMF factorization results in one network per subject, condition, and component. APB abductor pollicis brevis, FDS flexor digitorum superficialis, Tz trapezius, RSM right sensorimotor bipolar channel, LSM left sensorimotor bipolar channel

Group-level brain-muscle networks (BMN) were obtained by decomposing the coherence spectra of the 36 pairs of channels (2 EEG and 7 EMG channels), conditions, and subjects using non-negative matrix factorization (NMF). Reconstruction quality was assessed by increasing the number of components and evaluating the percentage of Frobenius norm of the coherence spectra accumulated by the components. The final number of components corresponds to the number for which a subsequent increase in one component results in less than a 2% increase in the variance accounted for. The decomposition resulted in two matrices, one corresponding to frequency components and one to the weights for the different components. The weights matrix can be represented as frequency-specific networks for each condition and subject where the strength of connectivity for each pair of channels gives the edges of each network. In order to obtain subject-level brain-muscle networks, NMF was applied to the sub-epochs coherence spectra and the above procedure was carried out to select the number of components (Fig. 2). The visualization and analysis of the resulting networks was conducted with NetworkX [106].

### Event-related synchronization/desynchronization

To obtain ERD/S, EEG was 0.5 Hz high-pass filtered (one-pass zero-phase FIR filter with a length of 1651 samples) and current source density transformation based on spherical splice surface Laplacian [107] was applied to reduce volume conduction and to obtain less correlated sensors [108]. In order to determine subject-specific frequency bands, we used FOOOF [109] to parameterize the power spectrum of each subject across all trials and obtain the peak frequencies for mu-alpha and mu-beta bands. The bands were defined as a frequency window of ± 3 Hz centered at the peak frequencies. Data was epoched from − 3 s before movement to 3 s after stop command (− 3 to 18 s), and the epoch from 0 to 18 s was divided into equally spaced 3 s sub-epochs. Sub-epochs were band-passed according to the subject-specific frequency bands, Hilbert transformed and the absolute value of the complex signal was obtained for each sub-epoch. Baseline correction was applied by subtracting the mean activity of the baseline period (− 0.5 to 0 s) from each sub-epoch and dividing by the same value. Finally, ERD/S for each sub-epoch was computed as the average power from 0 to 3 s for each frequency band.

### Heart activity

Raw data for the difference between the channel placed on the left and right collarbone was processed with Neurokit2 0.2.0 toolbox [110]. Data was filtered with a 0.5 Hz high-pass Butterworth filter (order = 5) and a 50 Hz Butterworth notch filter (order = 2). R peaks in each 15 s epoch were detected using the method “neurokit.” Wrongly detected peaks were corrected by setting a specific R peak threshold for each subject. The HR was computed as the inverse of the average difference between consecutive R peaks (RR intervals). HRV was measured as the mean root square of successive differences between RR intervals. For each participant, a value was considered an outlier and discarded if it is below or above 3 standard deviations.

### First-stage statistical analysis

For each trial (and subject), a functional brain-muscle network was constructed by computing the average coherence in the range from 0.5 to 40 Hz for each pair of channels, Σ_ij_. Once the coherence matrix, Σ, is computed, the functional connectivity was studied by transforming Σ into binary matrices or networks, G. Two criteria for this transformation were used: a fixed correlation threshold and a fixed number of links criterion. In the first criterion, the matrix is thresholded by a value ρ giving networks with varying numbers of links. In the second, a fixed number of link criteria is established and therefore a specific coherence threshold is computed for each subject. For both criteria, the networks obtained were analyzed by a recently developed test [111] for determining if the mean network is the same for different groups or conditions. This statistical test is an ANOVA test developed for input data that are networks. We used the ANOVA test for networks for comparing the three conditions of imagery and the resting state condition. All tests will be implemented at the individual level. Suppose we analyze a single subject who has performed k_i_ trials in the imagery conditions, I, and k_r_ trials in the resting state conditions. For a given network construction criteria, we obtain the networks G_1_^i^, G_2_^i^, …, G_ki_^i^, G_1_^r^, G_2_^r^, …, G_kr_^r^, where G_i_^L^ represents the network of the trial j of the condition L now we can compute the value T defined in [111], and shown below:

$$T = \frac{\sqrt{2}}{a} \sum\limits_{L=\{i,r\}} \sqrt{k_{L}} \left( \frac{k_{L}}{k_{L}-1} \bar{d}_{G^{L}} \left(M_{L}\right) - \frac{k_{i} + k_{r}}{k_{i} + k_{r} -1} \bar{d}_{G} \left(M_{L}\right) \right)$$

where ${\underline{d}}_{{G}^{L}}\left({M}_{L}\right)$ (${\underline{d}}_{G}\left({M}_{L}\right)$) represents the average distance of the sample of networks of the condition L, G^L^ (from the pooled sample, G) around the average weighted network of condition L. See [111] for details. This statistic T verifies that the more negative T is, the greater the difference between conditions. Then, for each subject, we compute the value of the statistic T (and the *p* value) defined in [111] for comparing networks. Smaller negative values of T indicate that the imagery network and the resting-state network have larger differences from each other. Based on this statistic, we (1) determine which subjects present significant differences between their imagery network and the resting-state network, (2) rank the subjects according to the level of these differences, (3) estimate the sensitivity of our method for detecting functional network differences based on imagery when compared with resting-state in healthy subjects, and (4) identify the subnetwork that expresses the greatest differences between the conditions. The latter is done by minimizing T by brute force considering the different possible subnetworks (see (111) for more details). Moreover, for each subject, the median difference of the heart rate in the imagery condition and the resting state condition is computed, and the subjects are ranked by this median value. Finally, we compute Spearman (or in this case equivalent to Pearson) correlation between the ranks given by the heart rate and the ranks given by the functional networks explained before. The same analysis is performed for the motor execution condition, and to analyze the robustness of the results we performed the statistical test mentioned above for different thresholds and a statistic that combines these results (W in [111]) was computed.

### Second-stage statistical analysis

(A1) Edge weights node normalization was carried out for each subject and condition by dividing all networks’ edge weights by the maximum edge weight across all the frequency networks. The strength of the nodes in each network and condition were computed by summing the weights of the connecting edges. We compared the strength of each node in each network for the following contrasts: RS and motor imagery (MI) of hand movement, RS and MI of foot movement, and RS and MI of simultaneous hand and foot movement. Therefore, 27 tests were carried out (3 comparisons for each of the 7 EMG and 2 EEG channels). Two-tailed *t*-tests were used to evaluate each contrast. FDR correction was applied to the resulting *p* values.

(A2) Cardiac activity modulations by motor imagery were evaluated by fitting linear mixed-effects models to heart rate and heart rate variability with condition (motor imagery − resting state) as a fixed factor and subject as a random effect using R [112]. The normality of the residuals was with a Shapiro–Wilk test, when normality was violated transformations on the data were applied.

(A3) Cluster-based permutation analyses were used to assess the group effect of motor imagery on mu-alpha and mu-beta bands. The mean power in the canonical mu-alpha and mu-beta bands were obtained for each participant during the resting state, motor imagery, and motor execution conditions. Differences between conditions are analyzed as follows: (i) subject average for motor imagery trials is subtracted from the subject average for resting state trials (or from the motor execution trials), (ii) a one-sample *t*-test is performed on every channel, (iii) *t* values that exceed a dependent samples *t*-test threshold corresponding to an alpha level (*p* value) of 0.025 (two-tailed, number of observations = number of subjects) are clustered according to spatial proximity. The adjacency matrix for a Biosemi 64-channel layout as defined in Fieldtrip is used. (iv) *t* values for each electrode within each cluster are summed in order to obtain a summed *t* statistic per cluster (*t*-sum), (v) 2000 permutations of the data are computed, and for each permutation, the cluster with the biggest-summed *t* statistic is kept in order to obtain a null hypothesis distribution, and (vi) the proportion of clusters from the null hypothesis with more extreme values than the cluster obtained from the observed data yields the *p* value for a given cluster. We considered the critical cluster *α* level here to be 0.025.

(A4) We conducted Shapiro–Wilk to test whether the kinesthetic and visual imagery scores across subjects, as well as the subject average power for mu-alpha and mu-beta bands, are normally distributed. We use Pearson’s correlations when normality is met, to correlate subjects’ scores to power. Otherwise, Spearman correlations are used.

(A5) Subject-level classifiers were constructed to distinguish between imagery and resting state conditions using Scikit-learn v1.0.2 [113]. The observations correspond to the sub-epochs for the imagery trials (~ 360) and the sub-epochs for the resting state trials (~ 360). The features are the weights for each component for each channel pair obtained from the spectral coherence decomposition (brain-muscle networks, BMN), the average heart rate and heart rate variability (heart activity features), and the power for the mu-alpha and mu-beta band during the sub-epoch, and immediately after the stop command. Random forest and SVM classifiers were implemented using a stratified group tenfold cross-validation procedure, where on each fold all sub-epochs from the same trial were grouped together in the train or test data set. The mean accuracy across folds was computed. This procedure was repeated 100 times yielding 100 accuracies per classifier. To test the significance of the classifiers’ accuracies, we followed standard practice [114] by evaluating the classifier performance using a non-parametric statistical approach. The labels for the observations were randomly permuted 1000 times and for each permutation, the classifier accuracy was obtained. We compared the mean accuracy of our original data against the empirical null distribution of classification accuracies. The proportion of null classification accuracies that are greater than the AUC of the original data yielded our *p* values. We used recursive feature elimination to evaluate the impact of the brain-muscle features on the classifiers’ accuracies. Moreover, if classification is successful we will proceed to evaluate the impact of the number of observations on the classifiers’ accuracy to determine whether task length could be reduced. Short tasks are preferable to avoid patient fatigue and facilitate their implementation in the medical environment. For this, we will remove observations in 10% steps, in a balanced manner across conditions, and re-run the classification.

The level of significance is established at *α* = 0.05 for all the proposed statistical tests.

### Number of participants and power analysis

**Fig. 3:**
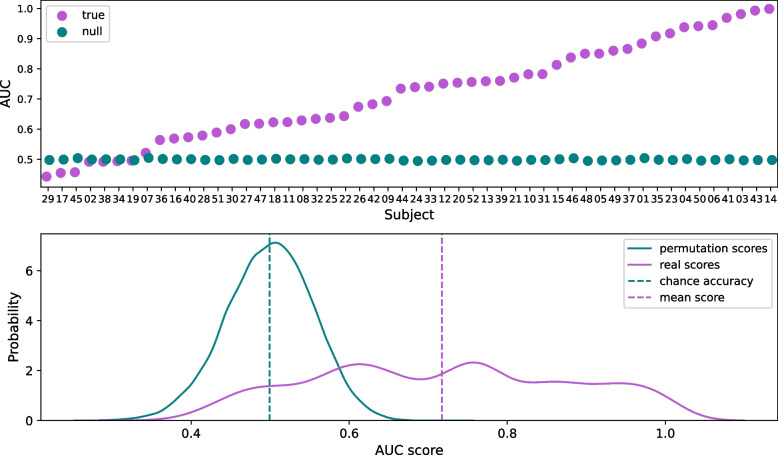
Motor imagery of left-hand versus right-hand classification performance across subjects. Top. Mean area under the curve score for each participant (purple). Mean area under the curve for 500 classification accuracies after randomly permutating the trial labels (green). Bottom. Kernel density estimation for the null distribution of AUCs (green) and for the original data (purple) across subjects

The main objective of our study is to detect covert command-following at the individual level by combining multimodal information. In healthy participants, motor execution is not impaired, and moving elicits overt behavior easily detected with EEG and EMG. In this study, we consider healthy participants imagining movement as a model of unresponsive patients trying to execute a movement. Motor imagery measured with EEG shows intersubject variability in healthy participants, indeed, brain-computer interface literature estimates that 10–30% of people are not able to willfully modify their brain activity by attempting motor imagery [115–117]. This estimate is likely influenced by differences in individual abilities, training, task, and analysis pipelines. The sampling size of this study has to guarantee that we find participants that are able to perform motor imagery to test the proposed brain-muscle network approach. To address this, we developed a motor imagery classification pipeline based on common spatial filters and linear discriminant analysis (LDA), a usual approach in brain-computer interface [118], that will be used to determine whether a participant is able to elicit motor imagery. In order to test this pipeline and estimate its power for classifying participants, we used a public dataset [119, 120] in which, in a single session and without previous training, 52 subjects performed 100 trials of imagery of the left hand and 100 trials of imagery of the right hand. The data consisted of segments of − 2:5 s time-locked to the imagery cue. A 0.5 Hz high-pass filter (one-pass zero-phase FIR filter with length 3381 samples) was applied to remove slow drifts, followed by a 7–40 Hz band-pass filter (one-pass zero-phase FIR filter with length 845 samples). The epochs were cropped from 0.5 to 2.5 s and as features, we used 4 spatial filters of the common spatial pattern (CSP) using MNE [99]. A LDA classifier was implemented for each participant’s data using a stratified tenfold cross-validation procedure and the mean accuracy, measured as the area under the curve (AUC), across folds was obtained. This procedure was repeated 50 times yielding 50 accuracies per classifier. To test the significance of the classifiers’ accuracies, we used a non-parametric approach. The labels for the observations were randomly permuted 500 times and for each permutation, the classification was recomputed. The proportion of null classification accuracies that have higher accuracy than the mean AUC for the original data resulted in the *p* value. The level of significance was established at *α* = 0.05. Forty participants were classified above chance with an overall AUC of 0.72 ± 0.16 (Fig. 3), which is consistent with the population estimates.

Given this result in order to obtain at least 20 participants showing motor imagery as detected by our classifier, we would have to test at least 26 participants.

Importantly, this estimated sample size corresponds to distinguishing different types of imagery, specifically right from left-hand imagery, which has proven difficult as some subjects show poor cortical lateralization [121]. In our analysis, we will try to distinguish trials in which a participant is carrying motor imagery from resting state trials; our effect size is expected to be bigger than the one associated with the analyzed dataset, so we argue that the sample size is a conservative estimate of the one needed for this study. We propose an initial sample size of 35 participants, in the event that we reach a subject-level classification above chance using CSP information for 20 participants, data collection will be stopped before completing the proposed sample size. The custom code for this analysis can be found at [122].

### Participants and data replacement

The sample consisted of right-handed healthy individuals without neurophysiological or musculoskeletal disorders between the ages of 18–45 years old. They were informed about the experimental protocol and objectives, and written consent was provided. We replaced a given participant if they did not complete any part of the study.

### Predicted outcomes

We expect to find brain responses consistent with motor imagery for at least half of the participants tested; this would be reflected in the number of participants for which the motor imagery classifier is able to distinguish imagery and resting trials.

Trials during motor imagery should elicit an increase in HR and HRV in comparison to resting state; the result of the linear mixed-effects models will provide information on this.

Although we expect a greater correlation between a participant’s score in the kinesthetic subscale of the MIQ-RS with the power for mu bands during motor imagery than for the visual subscale, the evidence regarding the predictive value of MIQ-RS on participants’ motor imagery abilities is not robust [123].

At the group level, we expect the motor imagery conditions to elicit an increase in the strength of nodes associated with the specific muscles involved in the imagined movement in comparison to resting state. During imagined movement of the hand, we expect the nodes corresponding to hand (abductor pollicis brevis) and arm (flexor digitorum superficialis, extensor digitorum, biceps brachii) muscles to show an increase in strength, compared to resting sate condition. During imagined movement of the foot, we expect the nodes gastrocnemius mediale and tibialis anterior to exhibit an increase in node strength, compared to the resting state condition. Motor imagery should elicit an event-related desynchronization for the mu-alpha and mu-beta bands in comparison to resting state, that is, a reduction in power should be observed during the mental representation of movement.

For participants for which motor imagery was detected using the CSP-based classifier, we expect to distinguish resting state from motor imagery with the classifier described in A5. In particular, we expect that the brain-muscle network information results in an improvement in classification accuracy compared to classifiers based only on power modulations.

## Results

A total of 38 participants took part in the study. During one session, EEG collection could not be carried out due to a technical issue and the participant was removed from the study. Two participants did not manage to finish the task and were also discarded. The final sample consisted of 35 participants (21 female, age = 25.6 ± 6.0).

### Initial motor imagery classification

**Fig. 4:**
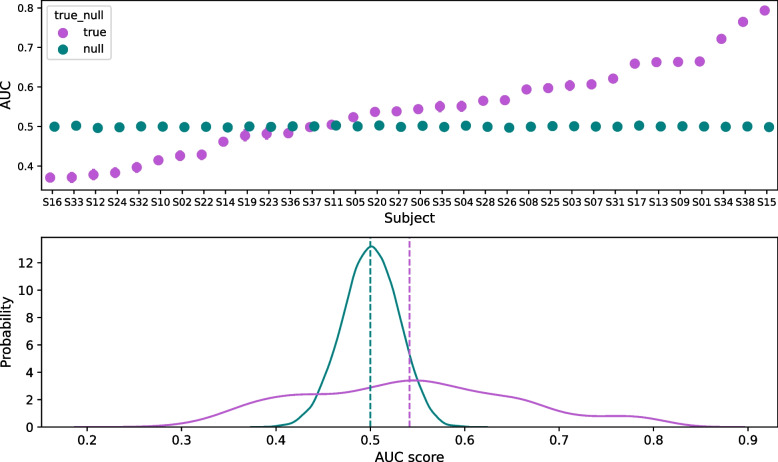
Motor imagery versus resting state performance across subjects for the LDA classifier based on CSP. Top. Mean area under the curve score for each participant (purple). Mean area under the curve for 500 classification accuracies after randomly permutating the trial labels (green). Bottom. Kernel density estimation for the null distribution of AUCs (green) and the original data (purple) across subjects

Running the LDA classifier based on common spatial patterns to discriminate between trials of imagery and trials of rest yielded low AUC values in comparison to the open data set used to estimate our sample size. Fifteen participants out of 35 were classified above chance (for one participant the classifier did not converge), with an overall AUC of 0.54 ± 0.11 (Fig. 4). We selected these 15 participants for the subsequent analysis.

#### First-stage statistical analysis

**Fig. 5:**
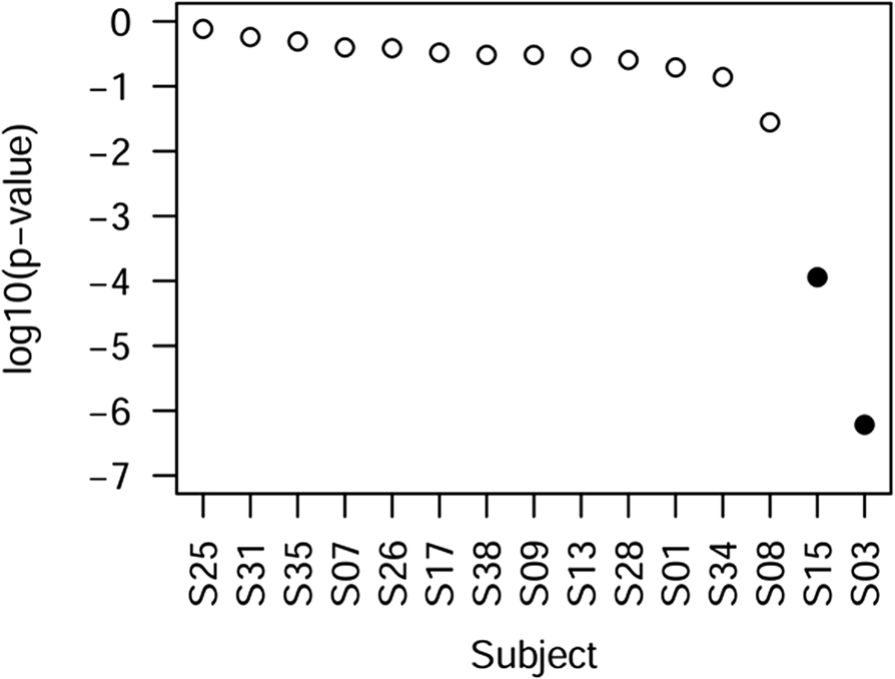
Comparison between imagery and resting functional networks. Black points correspond to subjects that present significant differences between both conditions

Imagery brain networks are compared to the resting state networks using the test proposed for each subject. Only two subjects show differences at the 0.05 significance level when corrected for multiple comparisons. The comparison between both conditions was performed across 14 different network groups: 7 constructed based on the criterion of maintaining a fixed number of links, and 7 based on establishing a link when the correlation exceeded a specific threshold. Figure 5 shows the median *p* value for each subject. Using the Benjamini–Hochberg procedure for multiple comparisons, we can ensure that subject 03 and subject 15 have different mean networks between conditions.

Finally, we compute Spearman correlation between the ranks given by the heart rate and the ranks given by the functional networks. Specifically, a *z*-score for heart rate was calculated by comparing the imagery condition to the resting condition, and the rank correlation between this *z*-score and the *t*-statistic of the functional networks was calculated. A high absolute value of the correlation coefficient suggests a degree of dependence between heart rate and the functional networks. However, the correlation coefficient from this comparison was not significantly different from zero (*p* value = 0.37).

### Second-stage statistical analysis

#### Brain-muscle networks are not affected by motor imagery.

**Fig. 6:**
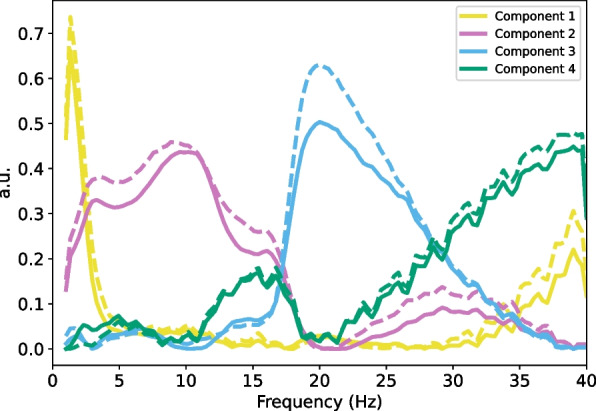
Frequency spectra of the four components obtained using NMF. Dashed lines correspond to the decomposition of the coherence spectra for the motor imagery and resting state trials. Continuous lines correspond to the decomposition of the coherence spectra for the motor execution and resting state conditions

**Fig. 7:**
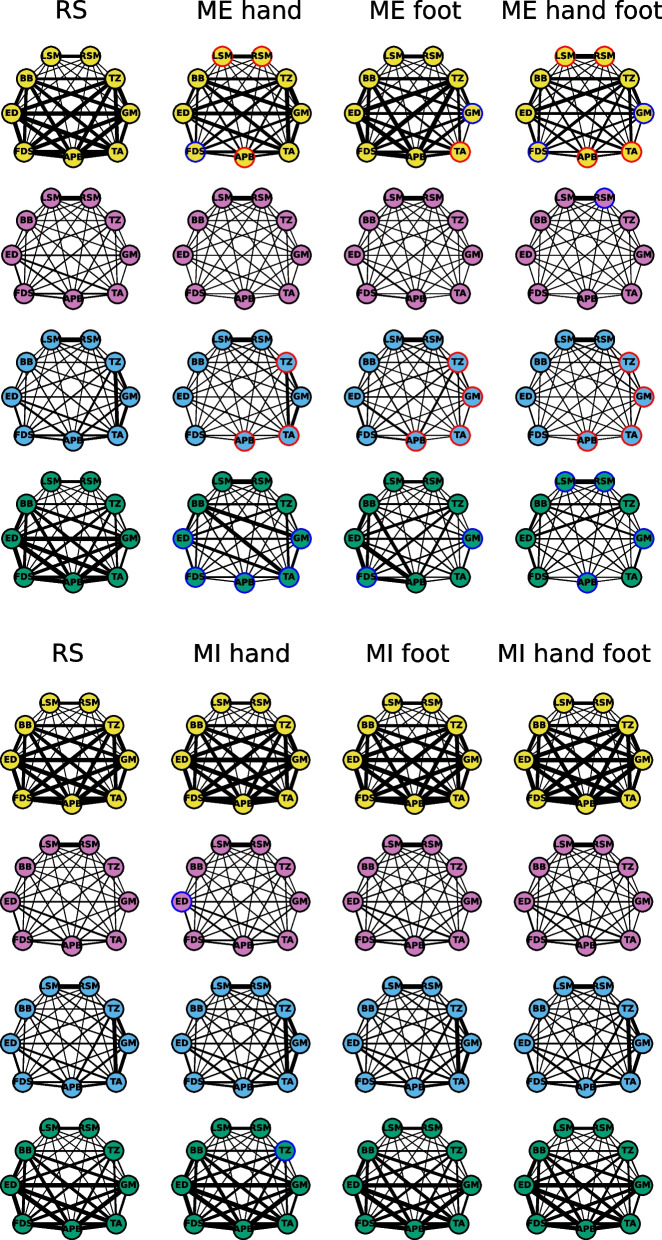
Functional brain-muscle networks. Top. Networks during motor execution (ME). Bottom. Networks during motor imagery (MI). Each node’s strength was contrasted to the strength of the same node during resting state (RS) (leftmost network). Red circles indicate significant differences after FDR correction, blue circles mark significant nodes before FDR correction, and black circles denote no difference to resting state node strength. The thickness of the edges corresponds to the strength of the connections between nodes

Functional brain-muscle networks were constructed by applying NMF to the magnitude squared coherence computed for pairs of channels during the resting state and motor imagery conditions. Reconstruction quality assessed with the Frobenius norm showed that 4 components accounted for 89.98% of the variance of the MSC matrix, and computing the decomposition for more components resulted in improvements smaller than the 2% established as threshold (3 components: 84.33%, 5 components: 91.34%). The decomposition yielded four separate frequency components (component 1, 0 to 5 Hz; component 2, 5 to 15 Hz; component 3, 15 to 30 Hz; component 4, 30 to 40 Hz; Fig. 6), which can be considered as four frequency-specific networks, consistent with previous research [93]. For each network and motor imagery condition, the node strength was contrasted to the node strength during resting state. No significant differences were obtained after FDR correction (Fig. 7, S2, S3).

In order to explore the functional brain-muscle networks during motor execution, the same procedure was carried out but considering motor execution and resting state trials. The factorization in 4 networks resulted in an accounted variance of 88% (Fig. 6). The network for component 1 associated to the coherence for lower frequencies showed a behavior that was consistent with a decrease in node strength for muscles involved in the different movement conditions. A decrease in node strength was observed for APB, LSM, and RSM during hand-moving trials, while during foot-moving trials node strength for TA was reduced. Finally, during simultaneous movement of hand and foot, node strength for APB, TA, RSM, and LSM was smaller than during resting state (*p* values < 0.043). For the third frequency network (15 to 30 Hz), simultaneous movement of hand and foot, as well as foot movement, elicited a decrease in connectivity for TZ, GM, TA, and APB (*p* values < 0.047). Hand movement showed a decrease in strength for nodes APB, TA, and TZ (*p* values < 0.042). No significant differences were observed for the networks for components 2 and 4 (Fig. 7).

#### Group analysis did not reveal differences in heart activity during motor imagery

Linear mixed models were implemented to test whether heart activity was different between motor imagery and resting state trials. Residuals for both heart rate and heart rate variability models violated normality, and logarithmic and inverse data transformations did not improve this. The models evaluated on the original data showed that the experimental condition did not explain the variability observed in heart rate (*β* = 0.01, *p* = 0.98, *R*^2^ = 0) and heart rate variability (*β* = 4.22, *p* < 0.001, *R*^2^ = 0). Similar results were obtained for models on transformed data.

#### Motor imagery and motor execution produced modulations of the sensorimotor rhythms

**Fig. 8:**
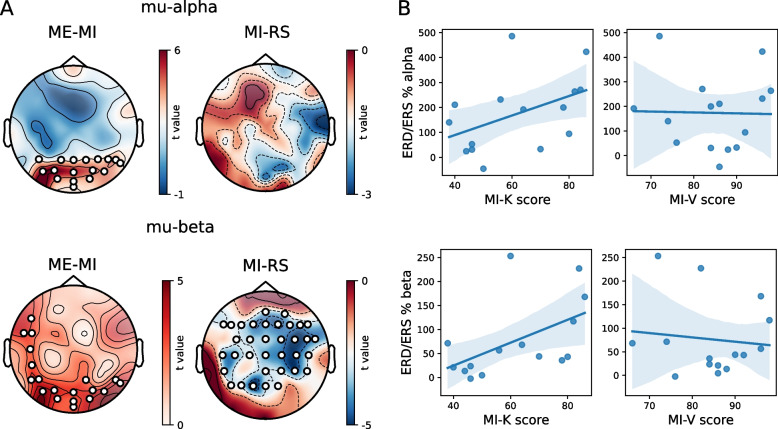
**A** Cluster-based permutation analysis for the mu-alpha power (top) and the mu-beta power bands (bottom). Left: *t* values for the contrast between motor execution (ME) and motor imagery (MI). Right: *t* values for the contrast between motor imagery and resting state (RS). **B** Top. Pearson correlations between kinesthetic motor imagery scores (MI-K) or visual imagery scores (MI-V), and the ERD/ERS for the mu-alpha power. Bottom. Correlation between motor imagery scores and the ERD/ERS for the mu-beta power

Cluster permutation analysis yielded significant differences between motor imagery and resting state for the mu-beta band. A cluster comprised of 35 centro-parietal electrodes showed lower mu-power during motor imagery compared to resting state trials (tsum = − 129, *p* = 0.005, Fig. 8A). In addition, motor execution showed increased power in the mu-beta band compared to motor imagery (tsum = 62, *p* = 0.0015, Fig. 8A) in parietal and occipital electrodes. Finally, power for the mu-alpha band was increased for motor execution in contrast to motor imagery in occipital electrodes (tsum = 67, *p* = 0.0015, Fig. 8A).

#### Kinesthetic motor imagery correlates with power modulations

The motor imagery scale showed an overall score of 73.13 ± 11.3, with a lower score for kinesthetic motor imagery (30.8 ± 8.6) than for visual motor imagery (42.3 ± 4.7) (paired-samples *t*-test *t*(14) = − 5.5, *p* = 7.8e − 5). Normality tests showed that visual scores (*z* = 0.74, *p* = 0.69) and kinesthetic scores (*z* = 5.5, *p* = 0.065) were normally distributed. Average ERD/S values were obtained for each subject by averaging the values for the electrodes taking part in the cluster yielded by the cluster permutation analysis for the mu-beta band. The ERD/S values for the mu-beta band (*z* = 5.6, *p* = 0.060) and the mu-alpha band (*z* = 1.31, *p* = 0.52) were normally distributed. For the mu-beta band, a small correlation was found between the kinesthetic imagery scores and the ERD/S values (*r* = 0.51, *p* = 0.048). No correlation was found between the ERD/S and the visual imagery scores (*r* = − 0.11, *p* = 0.80). For the mu-alpha power, no correlation was found with the kinesthetic motor imagery scores (*r* = 0.45, *p* = 0.092), nor the visual imagery scores (*r* = − 0.02, *p* = 0.93) (Fig. 8B).

#### Cortical power and heart rate variability are markers of motor imagery

**Fig. 9:**
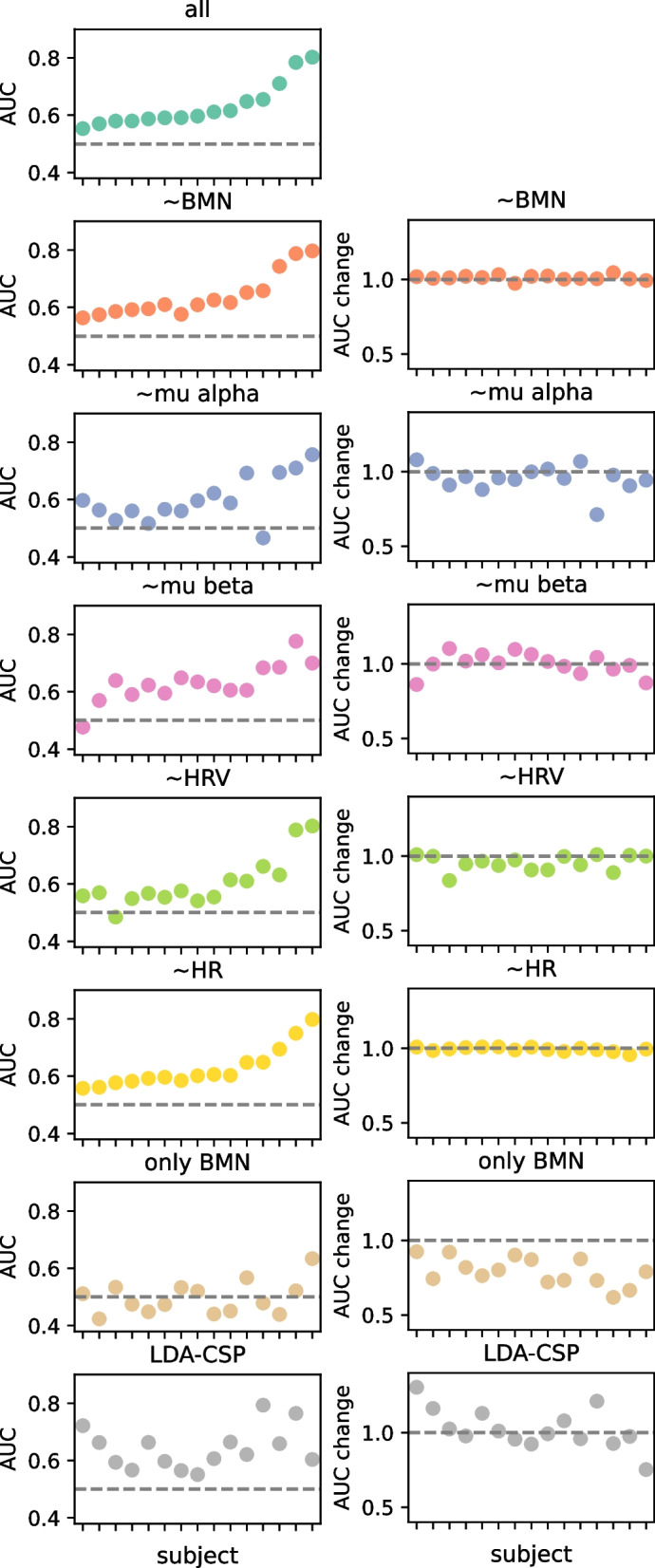
Subject-level classifiers based on brain-muscle networks, EEG power, and heart activity features. Left: average AUC across folds with 95% bootstrap confidence interval for each subject and classifier. Subjects are sorted considering the AUC for the full classifier. Right: ratio between the mean AUC for the classifiers lacking groups of features (or the LDA-CSP) and the mean AUC for the full classifier. ~ BMN: without the brain-muscle networks features, ~ HR: without the heart rate, ~ HRV: without heart variability, ~ mu-alpha: without the mu-alpha power for the 64 channels, ~ mu-beta: without the mu-beta power for the 64 channels, only BMN: classifier fed with only the brain-muscle networks features, and LDA-CSP: LDA classifier based on common spatial patterns

Subject-level random forest classifiers with decision trees as base estimators were implemented to distinguish between motor imagery and resting state trials. These classifiers were supplied with a set of features including the weights assigned to each channel pair by the NMF decomposition for the four frequency components (36 pairs of channels × 4 components), heart rate (HR), and heart rate variability (HRV) for the trial (see Fig. S1), and the power for mu-alpha and mu-beta for the 64 channels (totaling 274 features). The full-feature classifiers successfully classified all participants with an overall AUC of 0.63 ± 0.07, no different from the AUCs obtained for the LDA-CSP classifier for the same 15 participants (AUC = 0.64 ± 0.07, *t* = − 0.47, *p* = 0.648). We then systematically removed groups of features (BMN, HR, HRV, mu-beta, and mu-alpha) to evaluate their specific impact on classification and compared their performance to the full-feature classifiers. Removing the BMN features produced a slight increase in classification performance, with 15 participants classified above chance (AUC = 0.64 ± 0.07, paired *t*-test *t* = − 2.65, *p* = 0.019). Removing heart rate did not affect the overall (AUC = 0.63 ± 0.07, paired *t*-test *t* = 1.85, *p* = 0.086). However, excluding heart rate variability features led to a significant decrease in classification, with only 10 participants classified above chance (AUC = 0.60 ± 0.09, paired *t*-test *t* = 3.30, *p* = 0.005). Similarly, excluding mu-alpha power resulted in a reduction in the number of participants classified, without a significant change in the overall AUC (9 participants classified above chance, AUC = 0.60 ± 0.08, *t* = 2.10, *p* = 0.053). Removal of mu-beta features did not affect the overall AUC, but one participant could not be classified above chance (14 participants classified, AUC = 0.63 ± 0.07, *t* = 0.11, *p* = 0.91). Lastly, we implemented a classifier using only the BMN features to explore their potential to distinguish motor imagery from resting state trials. However, only one subject was classified using this approach (AUC = 0.50 ± 0.06, *t* = 7.34, *p* < 0.001) (Fig. 9). SVM classifiers with linear kernel functions were tested but failed to converge or produced unsuccessful models.

## Discussion

The aim of the present study was to evaluate the potential of a network approach to EMG and EEG recordings to detect covert command-following in healthy participants. Data from subjects that showed brain modulations consistent with motor imagery (MI) was further assessed by computing intermuscular coherence between muscles and scalp electrodes, EEG power for the mu bands was extracted, and heart activity was analyzed. Subject-level, as well as group analyses, were performed to evaluate the behavior of these variables during MI compared to the resting state.

The task was performed by 35 healthy participants. Fewer than half of the individuals exhibited brain activity consistent with motor imagery as indicated by the CSP-based classifier accuracies. The relatively low accuracy in classification could stem from multiple factors. In typical BCI experiments, motor imagery is elicited on cue and measured within the first seconds after the cue onset, as responses are higher closer to the initiation of the mental task [63, 124]. However, our study differed in that we assessed MI carried over extended periods during which participants repeatedly imagined the movements. The multiple initiations of motor imagery within a trial, coupled with the fact that the motor imagery trials consisted of different movements, likely introduced increased variability in our data resulting in a more challenging classification. Indeed, imagination of feet and hand movement has been shown to elicit different ERD/S profiles [125]. Furthermore, the extended duration of the task may have led to fatigue among participants, consequently impacting their performance as the task progressed. Although our task departs from the conventional motor imagery experiments, we argue that it represents a more ecological approach, particularly in light of its application to individuals with disorders of consciousness. The expectation that DoC patients can readily evoke motor imagery or motor execution immediately after cue can be unrealistic, and allowing the individuals to engage and repeat the rehearsal of movement freely could help capture temporally variable responses. In addition, a task with a similar design that instructs patients to execute hand movements has been successfully implemented to detect cognitive motor dissociation (CMD), patients who show a dissociation between behavior and brain response [33]. Importantly, the classification of 15 participants was successful and provided us with a sample over which to test the hypotheses of our study.

Kinesthetic motor imagery elicited a decrease in the mu-beta band compared to the resting state with a widespread distribution over central and parietal electrodes. Normally, MI in the mu-beta band elicits a contralateral ERD during imagined movement initiation [126, 127], nevertheless, modulations have also been observed in central [128] and ipsilateral regions [129]. Although no differences were obtained for the mu-alpha band at the group level, the subject-level analysis showed the relevance of the mu-alpha power to MI, as excluding this information hindered the classification for more than one-third of the participants. Importantly, in this analysis, the mu-alpha central frequency was determined individually for each participant, which probably enabled better results. One of the hypotheses of our work was that ERD/S in the mu-alpha and mu-beta bands would correlate with the subjective perception of a participant’s motor imagery capabilities as measured with the MIQ-RS. Consistent with previous research [130], we found a small correlation between the mu-beta power and the scores for the kinesthetic items of the scale, suggesting the notion that subjective kinesthetic motor imagery abilities can be informative on the level of cortical modulation during this type of motor imagery. MI and motor execution are believed to activate partially overlapping areas, with a weaker activation during MI. fMRI and PET studies consistently show an activation of the supplementary motor area during MI and motor execution [76, 77, 131–136], and several parietal areas are commonly activated as well [132, 137–139], with a controversial involvement of the primary motor cortex [61, 140]. In our study, ME and MI did not show differences in central regions but an increase in the mu-beta band in parietal electrodes was obtained, suggesting a more robust ERS during actual movement. In addition, occipital power was increased during motor execution for both mu power bands, which could be related to top-down inhibition of cortical areas irrelevant to the task [141], which has been reported for the mu-alpha band during repetitive movements [142]. In addition, greater activation of the occipitotemporal cortex has been observed during motor execution compared to motor imagery following a topographic representation of bodily parts [143].

The main hypothesis of our work was that the functional network configuration during motor imagery would be different from the resting state networks and that muscles activated during a specific motor imagery condition would be consistent with the muscles that are activated when the same movement is actually executed. Unfortunately, no differences were found in node strength for any of the muscles and motor imagery conditions. Moreover, classifiers built on only these features did not prove useful to distinguish motor imagery from resting state trials, and excluding the brain-muscle networks features from the subject-level classifiers yielded overall better performances. Overall, motor imagery did not elicit significant changes in the brain-muscle network configurations. Evidence of muscle activation during motor imagery is far from consistent, and it remains to be explained whether the contradictory results are related to intersubject variability [84, 144], or methodological differences such as signal processing, electrode placement, and task demands [145]. To our knowledge, no other work has tried to evaluate the coherence between EEG and EMG and intermuscular coherence during motor imagery using NMF at a group or subject-level. It is possible that the network approach followed here was not sensitive to capture changes in coherence. Nevertheless, an exploratory analysis to evaluate changes in EMG amplitude during motor imagery yielded no differences between any of the imagery conditions and the resting state condition (S4, see supplementary material), supporting the idea that motor imagery was not associated with muscle contractions in our participants. In the future, analysis could be carried out to detect changes in the EMG during motor imagery in comparison to resting state, by assessing changes in the mean and median values of the power spectra [146, 147].

During motor execution, a decrease in corticomuscular coherence in low frequencies (~ 5 Hz) between the sensorimotor cortices and the different muscles was observed as a reduction in node strength. This aligns with the findings for the gait cycle using a similar methodological approach [104]. Corticomuscular coherence for low frequencies was increased during the static phases of gait and reduced during movement. In our study, the reduction was only observed for motor conditions involving hand movement, possibly due to the hand’s broader cortical representation [148–150]. Additionally, we observed decreases in node strength for muscles involved in each movement for the 15–30 Hz component. Intermuscular coherence for this frequency band is typically decreased during the dynamic phase of movements [151]; this is consistent with evidence on peripheral and cortical rhythms showing a reduction in coupling between muscle and cortical activity during movement execution [40, 46, 151].

Supporting our second hypothesis, we show that bodily responses are informative of MI. Although we failed to model the effect of motor imagery on HR and HRV on grouped data, heart rate variability was modulated in a subject-specific manner during the mental rehearsal of movement, as these features were particularly relevant to distinguish MI from RS at the subject-level. HR acceleration has been shown during the first seconds of motor imagery initiation [152], and increases in heart rate have been reported during MI [153], particularly when imagined motor activity is perceived as effortful [68–70]. It has been posited that the brain constructs internal models of the environment as well as of our body and can access these models not only via action but also during mental tasks [154]. In our study, the direction of the effect of motor imagery on HRV was variable across subjects (Fig. S1). This could be linked to the individual mental effort elicited by the task [155], and it is possible that different patterns of sympathetic and parasympathetic control may be underlying the observed profiles. The intersubject variability in heart response could potentially explain previous findings where no discernible group differences were detected [156].

Finally, while the performance metrics of both the LDA-CSP-based classifier and the novel classifier proposed in this study showed no significant differences, subject-level variations in the AUC were observed. Notably, certain participants demonstrated enhanced classification efficacy with one classifier over the other. Hence, future investigations should focus on elucidating how to optimally combine the distinct information captured by the different approaches, improving overall classification accuracy and reliability.

Behavioral assessments to diagnose patients with disorders of consciousness rely on preserved motor functions and may fail to detect subtle motor responses [157, 158]. Complementary assessments based on neuroimaging typically try to bypass this limitation by demanding motor imagery from DoC patients [159]. Our aim was to contribute to the field by developing a new motor imagery task with the potential to detect command-following in DoC patients based on combined information from accessible tools. The task design was inspired by an assessment that is currently used in multiple centers to evaluate cognitive motor dissociation by asking patients to execute a movement for 10 s while employing spectral power markers to detect whether a patient was following the instructions [33, 159, 160]. As the EEG neural correlates of motor execution and motor imagery are similar [161], and some research using EMG has shown specific muscle activity when participants carry motor imagery [72], we considered that a healthy participant imagining simple movements could effectively model a DoC patient attempting to execute a movement without producing an overt response. While our results suggest against the immediate clinical deployment of our task, we are confident that our findings offer strong evidence supporting the use of bodily signals as a means to detect awareness. Integrating heart activity, measured through ECG recordings, into current clinical assessments could significantly enhance their efficacy. In the brain-computer interface literature, the potential of hybrid classifiers based on the combination of EEG and ECG information has been proposed [162], with some successful implementation for paradigms based on motor imagery [63] and selective attention [163]. This addition could be particularly straightforward for existing motor imagery evaluations or BCI setups used with patients suffering from DoC [164, 165], and also holds promise for tasks involving motor execution [30, 33].

Patients who suffer from disorders of consciousness are a heterogeneous group with severe brain injuries produced by diverse etiologies [166] who benefit from multiple and multimodal assessments [167, 168]. Some patients may be able to produce small motor responses and therefore motor execution assessments combining EEG, EMG, and ECG information would be crucial, whereas patients exhibiting a dissociation between motor planning and execution [133] may benefit from assessments based on motor imagery that integrate EEG and ECG to detect the willful modulation of brain and bodily responses.

### Limitations

Our work has methodological limitations that should be taken into consideration for future studies. Firstly, participants imagined movement freely during the 15 s of each trial, therefore the onset and offset of each instantiation were undetermined and the power changes observed at the group level are the result of the averaged activity in that time span. Together with the fact that simultaneous motor imagery conditions were jointly analyzed probably impacted our results, limiting the interpretation of the topographies for the mu power effects.

## Conclusions

Overall, while brain-muscle functional networks were not modulated by motor imagery of hand and foot movements, heart activity and cortical power were crucial to detect when a participant was mentally rehearsing a movement. Our work highlights the importance of combining EEG and peripheral measurements to detect command-following, which could be important for improving the detection of covert responses consistent with volition in unresponsive patients.

## Supplementary Information

Supplementary Material 1.

## Data Availability

The authors declare that the data and code supporting the findings of this study are available at [122].

## Abbreviations

ANS: Autonomic nervous system
APB: Abductor pollicis brevis
AUC: Area under the receiver operating characteristic curve
BB: Biceps brachii
CC: Cortical coherence
CMC: Corticomuscular coherence
CMD: Cognitive motor dissociation
CSP: Common spatial patterns
DoC: Disorders of consciousness
ECG: Electrocardiogram
ED: Extensor digitorum
EEG: Electroencephalogram
EMG: Electromyography
ERD: Event-related desynchronization
ERS: Event-related synchronization
FDS: Flexor digitorum superficialis
FMN: Functional muscle networks
fMRI: Functional magnetic resonance imaging
GM: Gastrocnemius mediale
ICA: Independent component analysis
IMC: Intermuscular coherence
LDA: Linear discriminant analysis
LSM: Left sensorimotor
MCS: Minimally conscious state
ME: Motor execution
MI: Motor imagery
MIQ-RS: Movement Imagery Questionnaire-Revised Second version
MSC: Magnitude squared coherence
NMF: Non-negative matrix factorization
RSM: Right sensorimotor
TA: Tibialis anterio
TZ: Trapezius
UWS: Unresponsive wakeful syndrome
VS: Vegetative state
