# Supplementary material for: Assessing brain-muscle networks during motor imagery to detect covert command-following
Source: BMC Med. 2025 Feb 6;23:68. doi: 10.1186/s12916-025-03846-0 (PMC11803995; doi:10.1186/s12916-025-03846-0)
Supplement: Supplementary file 1 — Supplementary Material 1. [file 12916_2025_3846_MOESM1_ESM.docx]

**Supplementary material**

Assessing Brain-Muscle Networks during Motor Imagery to Detect Covert Command-Following

**Author names and affiliations:** Fló, Emilia^1^; Fraiman, Daniel^2^ & Sitt, Jacobo^1^

1. Sorbonne Université, Institut du Cerveau - Paris Brain Institute - ICM, Inserm, CNRS, APHP, Hôpital de la Pitié Salpêtrière, Paris, France
2. Departamento de Matemática y Ciencias, Universidad de San Andrés, Buenos Aires, Argentina & CONICET, Buenos Aires, Argentina


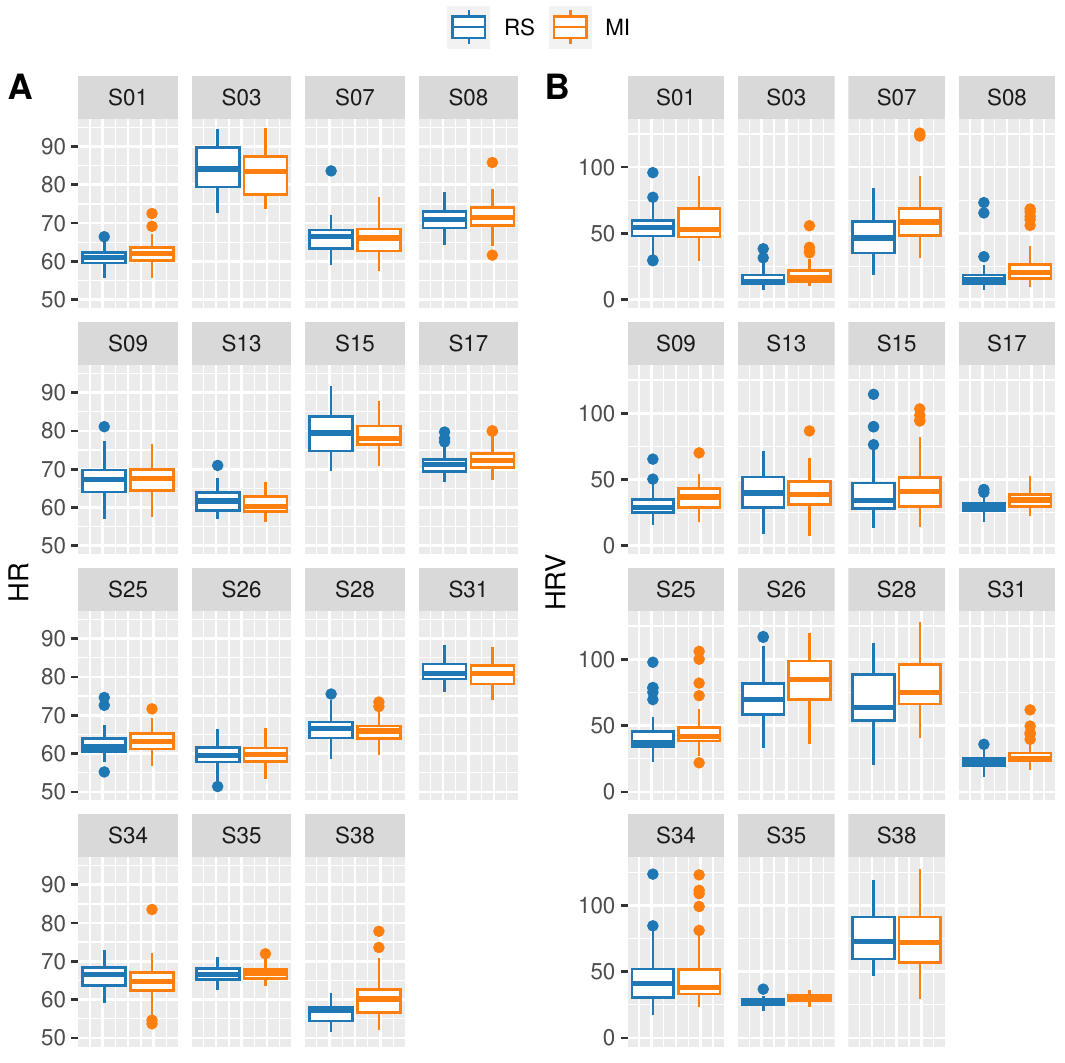


**Fig. S1**. Subject-level heart activity during motor imagery (MI) and resting state (RS)


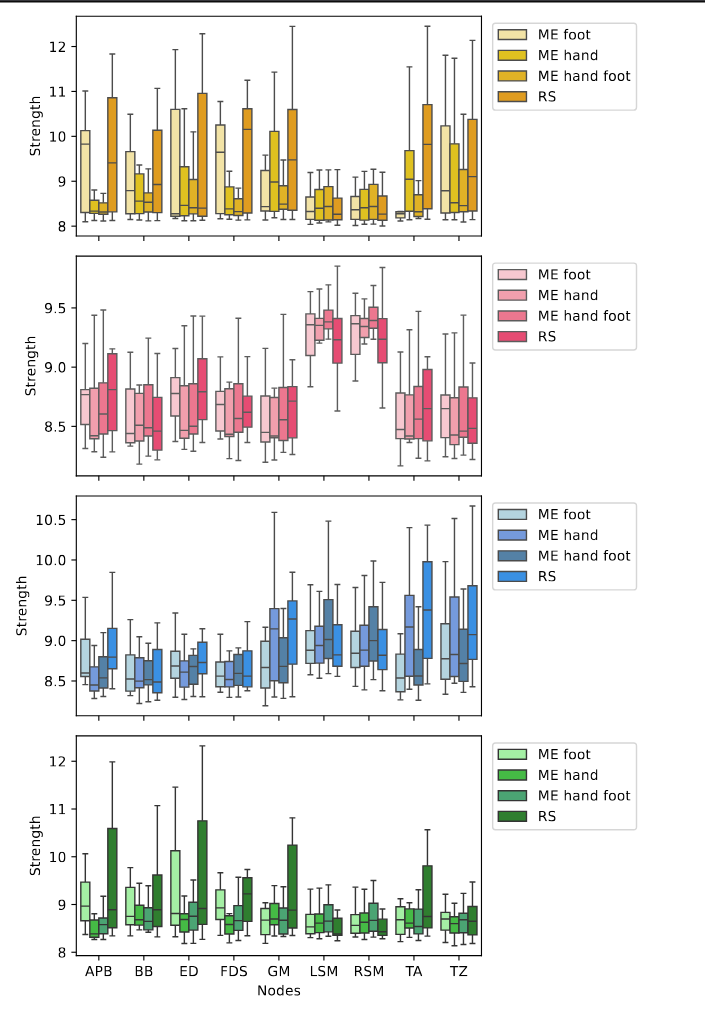


**Fig. S2**. Node strength for each brain-muscle network during resting state (RS) and motor execution (ME) conditions. Abductor policis brevis - APB, flexor digitorum superficialis - FDS, extensor digitorum - ED, biceps brachii - BB, gastrocnemius mediale - GM, tibialis anterior - TA, and trapezius - Tz.


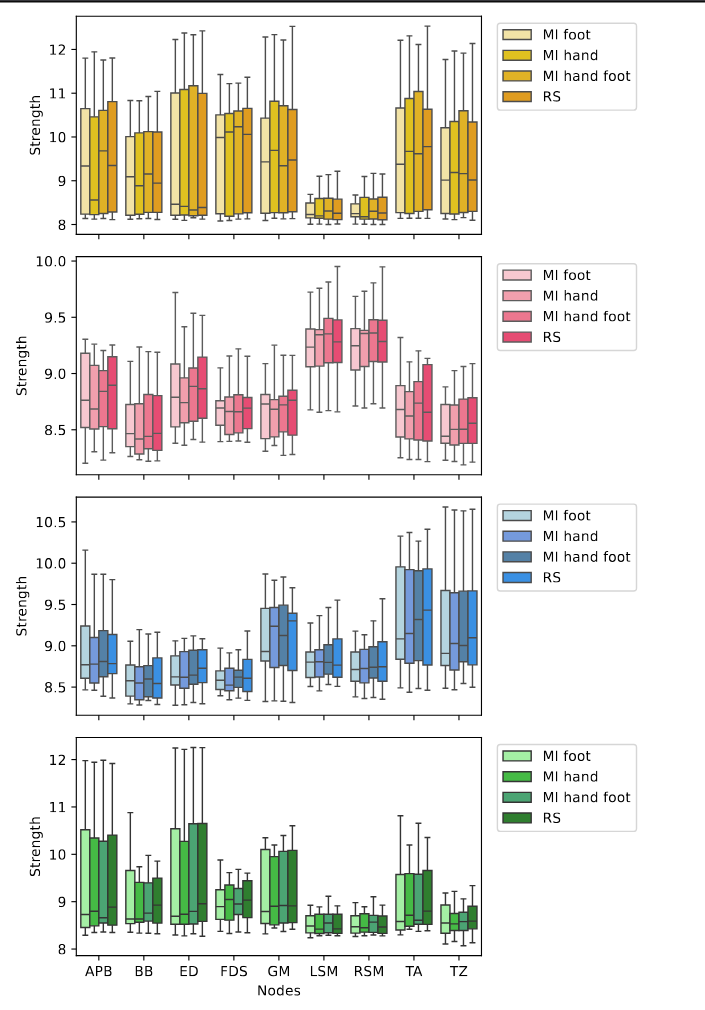


**Fig. S3**. Node strength for each brain-muscle network during resting state (RS) and motor imagery (MI) conditions. Abductor policis brevis - APB, flexor digitorum superficialis - FDS, extensor digitorum - ED, biceps brachii - BB, gastrocnemius mediale - GM, tibialis anterior - TA, and trapezius - Tz.

*Exploratory analysis*

For each subject, rectified EMG activity was averaged across time, condition, and electrode. The EMG amplitude for each muscle and imagery condition was statistically compared to the EMG activity for that muscle during the resting state with paired t-tests. For all comparisons the p values ≥ 0.066.


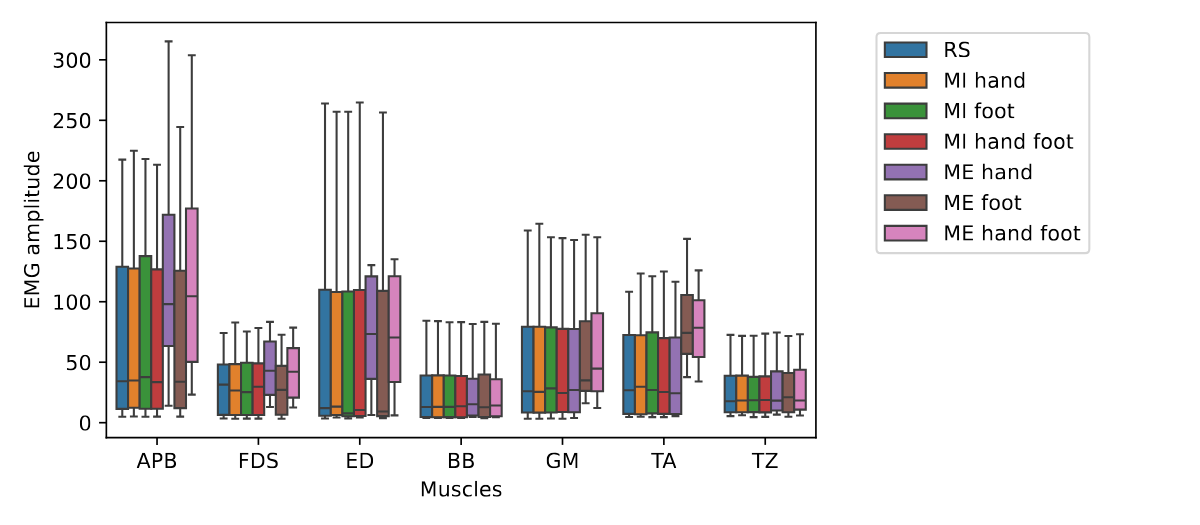
**Fig. S4**. EMG amplitude for each muscle during motor execution (ME), motor imagery (MI), and during resting state (RS) conditions. Abductor policis brevis - APB, flexor digitorum superficialis - FDS, extensor digitorum - ED, biceps brachii - BB, gastrocnemius mediale - GM, tibialis anterior - TA, and trapezius - Tz.
